# Performances of some low-cost counter electrode materials in CdS and CdSe quantum dot-sensitized solar cells

**DOI:** 10.1186/1556-276X-9-69

**Published:** 2014-02-10

**Authors:** Hieng Kiat Jun, Mohamed Abdul Careem, Abdul Kariem Arof

**Affiliations:** 1 Centre for Ionics University of Malaya (CIUM), Department of Physics, University of Malaya, Kuala Lumpur 50603, Malaysia

**Keywords:** Quantum dot-sensitized solar cell (QDSSC), CdS, CdSe, Successive ionic layer adsorption and reaction (SILAR), Counter electrode

## Abstract

Different counter electrode (CE) materials based on carbon and Cu_2_S were prepared for the application in CdS and CdSe quantum dot-sensitized solar cells (QDSSCs). The CEs were prepared using low-cost and facile methods. Platinum was used as the reference CE material to compare the performances of the other materials. While carbon-based materials produced the best solar cell performance in CdS QDSSCs, platinum and Cu_2_S were superior in CdSe QDSSCs. Different CE materials have different performance in the two types of QDSSCs employed due to the different type of sensitizers and composition of polysulfide electrolytes used. The poor performance of QDSSCs with some CE materials is largely due to the lower photocurrent density and open-circuit voltage. The electrochemical impedance spectroscopy performed on the cells showed that the poor-performing QDSSCs had higher charge-transfer resistances and CPE values at their CE/electrolyte interfaces.

## Background

As the world population grows, the demand for energy consumption will also increase in tandem. In order to meet the growing demand, there is a need to use renewable energy source as an alternative source for fossil fuels. One of the renewable energy routes is solar cells. Of all the solar cell technologies, quantum dot-sensitized solar cells (QDSSCs) have emerged as a widely researched topic in recent years [1-4]. The high interest in this field is due to the attractive properties of the quantum dots (QDs), namely ease of synthesis, ability to tune the band gap energy and possibility of attaining multiple exciton generation (MEG) [3-5]. Some examples of QDs include but not limited to Ag_2_S [6], CdS [7], CdSe [8], PbS [9] and CuInS_2_[10]. Recently, QDs based on organometallic perovskites such as CH_3_NH_3_Pbl_3_ have shown impressive efficiencies [11].

In QDSSCs, the working principle is almost similar to that of the dye-sensitized solar cell (DSSC) [12]. Upon light irradiation, the electrons in the QD will be excited into the conduction band (CB) leaving holes in the valence band (VB). The electrons will then get injected into the CB of the wide band gap semiconductor (usually TiO_2_), percolate through the TiO_2_ network and reach the substrate. The electrons reach the counter electrode (CE) by passing through the external load and reduce the redox mediators which donate electrons to fill the holes in the QDs. Thus, current is produced continuously as long as light is present without the consumption or production of any chemicals.

In order to obtain a high-performing QDSSC, material selection plays a major role [13]. The type of QD sensitizers, CE materials and electrolyte composition could affect the overall performance in one way or another. Among the prominent materials for QD sensitizers, CdS and CdSe are widely used due to their easy preparation. The QDSSCs based on them usually employ polysulfide-based liquid electrolytes. For CE, the usual choice is platinum even though other materials such as gold, Cu_2_S and reduced graphene oxide (RGO) are possible [14-16].

In this work, alternative low-cost CE materials were used in CdS and CdSe QDSSC assembly to understand the effect of CE materials towards the solar cell performance. The materials for the CEs used were commercially obtained or prepared economically at lab scale. Two different optimized polysulfide liquid electrolytes were used in the CdS and CdSe QDSSCs. Photoelectrochemical performance of the cells was investigated to assess the effect of the CE materials. The behaviour of the QDSSCs was also investigated using electrochemical impedance spectroscopy (EIS). This study was undertaken to explore the best low-cost and easy-to-prepare CE material for CdS and CdSe QDSSCs. To the author's best knowledge, there is no report in the literature on the performance of easy-to-prepare low-cost graphite, carbon soot and RGO used as CEs in QDSSCs.

## Methods

### Materials

Titanium dioxide (TiO_2_) paste (18NR) was obtained from JGC C&C, Kawasaki City, Kanagawa, Japan. Fluorine-doped tin oxide (FTO) conducting glasses (8 Ω/sq sheet resistance) purchased from Solaronix, Aubonne, Switzerland were used as electrode substrates. The di-isopropoxytitanum bis(acetylacetonate) needed for the TiO_2_ compact layer was procured from Sigma-Aldrich, St. Louis, MO, USA. Cadmium nitrate tetrahydrate, selenium dioxide, sodium borohydride, potassium chloride, sulfur and guanidine thiocyanate (GuSCN) were all purchased from Sigma-Aldrich while sodium sulfide nonahydrate was procured from Bendosen, Hamburg, Germany.

### Preparation of TiO_2_ film working electrode

A compact layer of TiO_2_ was first prepared by spin coating 0.38 M ethanolic solution of di-isopropoxytitanum bis(acetylacetonate) on the FTO surface of the substrate at 3,000 rpm for 10 s. The coated FTO glass was then sintered at 450°C for 30 min. The acquired TiO_2_ compact layer not only enhances the adhesion of TiO_2_ particles to the substrate but also provides a larger TiO_2_/FTO contact area ratio and minimizes electron recombination by reducing the contact between the electrolyte and the FTO surface [17]. The doctor blade method was used to spread the TiO_2_ paste on the compact layer in order to form the mesoporous network of TiO_2_. The newly deposited layer was also sintered at 450°C for 30 min in order to remove organic residues and moisture for obtaining a mesoporous TiO_2_ layer.

### Fabrication of CdS and CdSe QD-sensitized electrodes

Both CdS and CdSe QDs were prepared using the successive ionic layer adsorption and reaction (SILAR) deposition method. To fabricate CdS QDs, the TiO_2_-coated electrode was successively dipped into 0.1 M Cd(NO_3_)_2_ ethanolic solution for 5 min and into 0.1 M Na_2_S methanol solution for another 5 min. The electrode was rinsed with alcohol and allowed to dry in between the dipping process. This two-step dipping is considered as 1 SILAR cycle. Four SILAR cycles were used to prepare a CdS QD-sensitized TiO_2_ electrode.

For CdSe QDs, preparation process was performed in a glove box filled with argon gas [18]. TiO_2_-coated electrode was first dipped into 0.03 M Cd(NO_3_)_2_ ethanolic solution for 30 s followed by ethanol rinsing and drying. Then, it was dipped into Se^2-^ solution for 30 s followed by ethanol rinsing and drying. Se^2-^ solution was prepared by reacting 0.03 M SeO_2_ ethanolic solution with 0.06 M NaBH_4_. The mixture was stirred for about an hour before it was used for SILAR dipping process. Seven SILAR cycles were used to prepare a CdSe QD-sensitized TiO_2_ electrode.

### Preparation of CEs

Five types of CE materials were used: platinum, graphite, carbon, Cu_2_S and RGO. Platinum layer was prepared by spin coating a thin layer of commercial platinum solution (Plastisol from Solaronix) on the conducting glass surface and sintering at 450°C for 30 min. Graphite layer was obtained by rubbing pencil lead on the conducting glass surface. To obtain carbon layer, the conducting glass was placed over a candle flame for a few seconds so that black carbon soot formed readily on the surface. Cu_2_S electrode was prepared according to the procedure given in the literature [19]. In this procedure, a brass electrode was immersed in hydrochloric acid at 70°C for 5 min, and then, the treated brass was dipped into polysulfide aqueous solution containing 1 M Na_2_S and 1 M S for 10 min. Upon the solution treatment, Cu_2_S would be formed on the brass surface as a thin black layer. To prepare counter electrode with RGO, RGO powder (Timesnano) was mixed in the N-methyl-2-pyrrolidone (NMP) solution with 10 wt.% of polyvinylidene difluoride (PVDF). The suspension was then cast on the conducting glass and allowed to dry at 70°C.

### Assembly of QDSSCs

Solar cell was fabricated by clamping the QD-sensitized TiO_2_ electrode with a selected CE. Parafilm (130 μm thickness) was used as a spacer between the two electrodes. The spacer also prevented the liquid electrolyte from leaking. Prior to the cell assembly, few drops of polysulfide electrolyte were dropped onto the surface of QD-sensitized TiO_2_ film until the active surface area was covered with the electrolyte. Different polysulfide liquid electrolytes were selected for CdS and CdSe QDSSCs based on previous optimization reports [20,21]. The polysulfide electrolyte solution for CdS QDSSCs was prepared from 0.5 M Na_2_S, 2 M S and 0.2 M KCl in water/methanol = 3:7 (*v*/*v*) [20]. For CdSe QDSSCs, the polysulfide electrolyte contained 0.5 M Na_2_S, 0.1 M S and 0.05 M GuSCN in water/ethanol = 2:8 (*v/v*) [21]. An effective cell area of 0.25 cm^2^ was used for the solar cell performance investigations.

### Photoresponse and EIS measurements

Photocurrent-voltage (*I-V*) characteristics of the QDSSCs were measured using a Keithley 2400 electrometer (Cleveland, OH, USA) under illumination from a xenon lamp at the intensity of 1,000 W m^-2^. Efficiency was calculated from the equation

(1) $$\eta =\frac{J_{\mathrm{SC}}\times V_{\mathrm{OC}}\times \mathrm{FF}}{P_{\mathrm{in}}},$$

where *J*_SC_ is the short-circuit photocurrent density, *V*_OC_ is open-circuit voltage, FF is the fill factor and *P*_in_ is the intensity of the incident light. Measurement on each cell was repeated three times to ensure the consistency of the data.

The EIS study was performed using an Autolab potentiostat/galvanostat (Utrecht, The Netherlands). Measurement was performed on cells under dark and illuminated conditions. Light illumination was provided by a xenon lamp at the intensity of 1,000 W m^-2^. The EIS measurements were made on cells biased at potentials given and explained in the ‘Results and discussion’ section with a 15-mV RMS voltage perturbation in the frequency range 10^6^ to 0.01 Hz. EIS results were fitted with *ZSimWin* software to obtain the series resistance, *R*_S_ and charge-transfer resistance at the CE/electrolyte interface, *R*_CE_.

## Results and discussion

CdS and CdSe QDSSCs have been fabricated with QD-sensitized TiO_2_ layers prepared via SILAR method and selected liquid electrolytes. Both CdS and CdSe QD-sensitized TiO_2_ layers were assembled with the five different types of CE materials including platinum. The cell with platinum as the CE was used as the reference cell. The *J-V* curves for both types of QDSSCs showed that solar cell performance is considerably influenced by the choice of CE materials.

**Figure 1:**
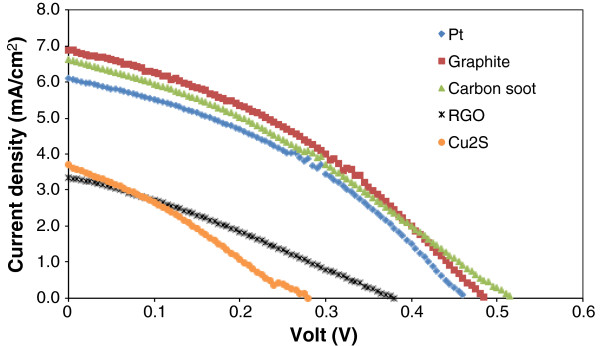
***J-V *** **curves of CdS-based QDSSCs with various CEs.**

For CdS QDSSCs, the *J-V* curves are shown in Figure 1 and the performance parameters are summarized in Table 1. Higher efficiencies of 1.06%, 1.20% and 1.16% are observed for solar cells assembled with commercial platinum catalyst, graphite layer and carbon soot, respectively, as CE materials. The solar cells with these CE materials produced current densities above 6.00 mA/cm^2^. These results indicate that carbon-based material (graphite and carbon soot) can be the alternative CE for CdS QDSSCs. On the other hand, Cu_2_S and RGO do not give better performances in our CdS QDSSC although better performances with these materials have been reported by other researchers with efficiencies above 3% [22,23]. The low performance of our QDSSCs with Cu_2_S and RGO as CEs is attributed to the respective overall low short-circuit current density, open-circuit voltage and fill factor. Nevertheless, the observed photocurrent density for the cell with Cu_2_S as CE is comparable with the published result of 3.06 mA/cm^2^[24]. In general, CdS QDSSCs exhibit low fill factors (less than 40%) with any of the tested CE materials.

**Table 1:** Performance parameters of CdS QDSSCs with various CEs

	***J***_**SC **_**(mA/cm**^**2**^**)**	***V***_**OC **_**(V)**	**FF (%)**	***η *** **(%)**
Pt	6.09	0.460	38	1.06
Graphite	6.89	0.485	36	1.20
Carbon soot	6.62	0.515	34	1.16
Cu_2_S	3.70	0.280	28	0.29
RGO	3.35	0.380	29	0.37

**Figure 2:**
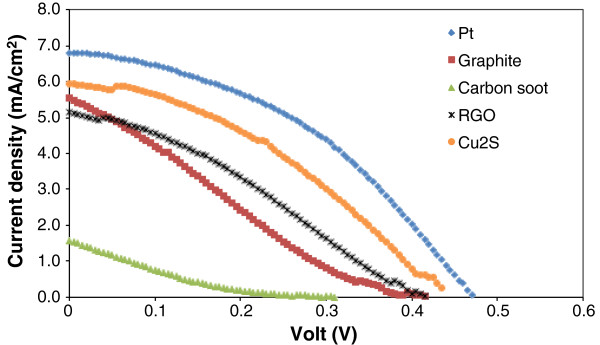
***J-V *** **curves of CdSe QDSSCs with various CEs.**

In the study of CdSe QDSSCs, *J-V* curves of each solar cell combination with different CE materials are shown in Figure 2, and the corresponding performance data are summarized in Table 2. Unlike the CdS QDSSC, the CdSe QDSSC exhibits high efficiencies with Cu_2_S and platinum as CE materials. Among these results, the best performance is observed in solar cell assembly with commercial platinum catalyst as the CE. The CdSe QDSSC with platinum as the CE produced an efficiency of 1.41% followed by 1.16% with Cu_2_S as the CE. The fill factor and *V*_OC_ with Cu_2_S are also good. These results show that Cu_2_S is compatible with CdSe QD as a CE material. On the other hand, carbon-based materials like graphite and carbon soot which work well in the CdS QDSSC perform poorly when coupled with CdSe QD-sensitized TiO_2_ electrodes. The poor performance from these materials could be attributed to the low electrocatalytic activity at the CE/electrolyte interface against the fast electron injection and transfer from CdSe QDs into the photoanode substrate. The preference of different CE materials for CdS and CdSe QD-sensitized TiO_2_ electrodes could be explained by electrochemical impedance spectroscopy (EIS) study. The observed performance of our QDSSC is rather low when compared with result from other groups. However, we anticipate the performance to be better if optimization of the photoanode is carried out such as addition of a scattering layer and passivation with a ZnS layer.

**Table 2:** CdSe QDSSC performance parameters with various CEs

	***J***_**SC **_**(mA/cm**^**2**^**)**	***V***_**OC **_**(V)**	**FF (%)**	***η *** **(%)**
Pt	6.80	0.470	44	1.41
Graphite	5.53	0.415	22	0.50
Carbon soot	1.58	0.310	15	0.07
Cu_2_S	6.01	0.430	45	1.16
RGO	5.15	0.415	31	0.66

**Figure 3:**
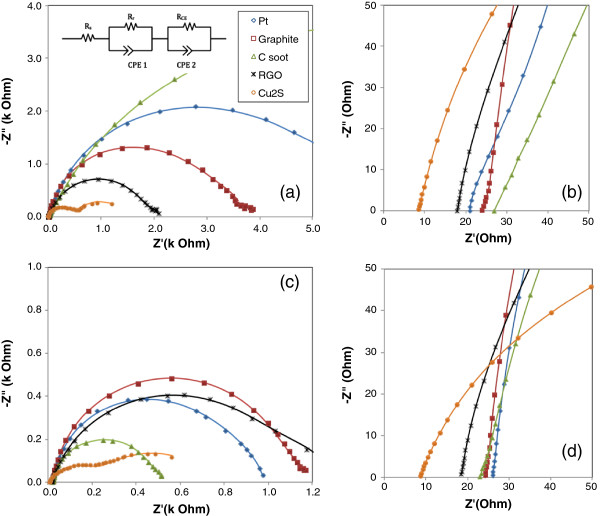
**Nyquist plots of CdS QDSSCs under dark condition and 1,000-W/m**^**2 **^**illumination. (a)** Nyquist plots of CdS QDSSCs in dark; the equivalent circuit of the QDSSC with the representation of impedance at CE/electrolyte interface (subscript CE), QD-sensitized TiO_2_/electrolyte (subscript r) and series resistance (subscript s). The symbol R and CPE denote the resistance and constant phase element, respectively. **(b)** Details of plots (a) at high frequencies. **(c)** Nyquist plots of the same cells under 1,000-W/m^2^ illumination. **(d)** Details of plots (c) at high frequencies. The solid lines are the fitted curves.

**Figure 4:**
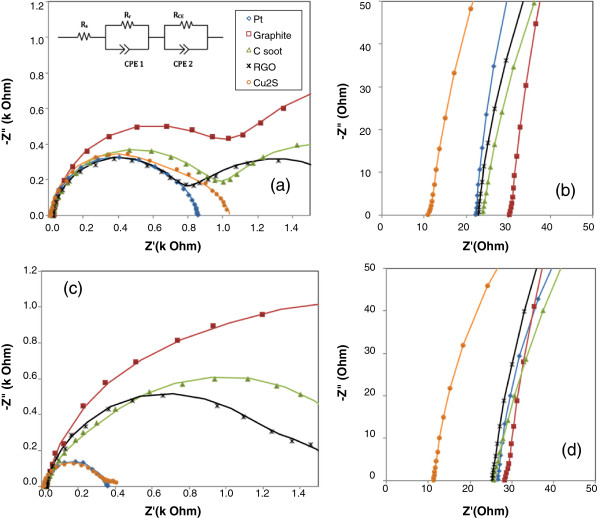
**Nyquist plots of CdSe QDSSCs under dark condition and 1,000-W/m**^**2 **^**illumination. (a)** Nyquist plots of CdSe QDSSCs in dark; the equivalent circuit of the QDSSC with the representation of impedance at CE/electrolyte interface (subscript CE), QD-sensitized TiO_2_/electrolyte (subscript r) and series resistance (subscripts). The symbol R and CPE denote the resistance and constant phase element, respectively. **(b)** Details of plots (a) at high frequencies. **(c)** Nyquist plots of the same cells under 1,000-W/m^2^ illumination. **(d)** Details of plots (c) at high frequencies. The solid lines are the fitted curves.

EIS is performed to understand the kinetic processes within the QDSSC. Typically, an EIS spectrum for a dye-sensitized solar cell (DSSC) consists of three semicircles in the Nyquist plot [25]. This characteristic is also applicable to QDSSC [24]. The three semicircles correspond to the response in high-frequency, intermediate-frequency and low-frequency regions when the cell is biased at its open-circuit potential. Response in the high-frequency region is attributed to the charge transfer between electrolyte and CE interface while the intermediate-frequency response denotes the electron transport in the QD-sensitized TiO_2_ layer and the recombination process at the QD-sensitized TiO_2_ and electrolyte interface. Finally, the low-frequency response relates to the diffusion process in the electrolyte. Generally, a double arc is observed for low-performing QDSSC where the feature of electrolyte diffusion is seldom present. In this study, the focus is on the first semicircle which is the response at high frequencies. Typically, the equivalent circuit of a QDSSC in a conductive state is a combination of a series resistance and two time constant elements as shown in the insets of Figures 3a and 4a [26]. The second time constant element represents the response of the CE/electrolyte interface.

The EIS investigations on CdS QDSSCs were performed at 0.45-V potential bias. This potential bias is selected at the median of the observed open-circuit voltage results. Meanwhile, for CdSe QDSSCs, the measurements were carried out at a bias of 0.40 V. Figure 3a shows the Nyquist plots of CdS QDSSCs having various CE materials under dark condition, and the details of the high-frequency responses are shown in Figure 3b. The response under dark condition serves as a reference for the responses under illumination (Figure 3c,d). The corresponding series resistance and charge-transfer resistance data obtained are tabulated in Table 3.

**Table 3:** EIS results of CdS QDSSCs

	***R***_**S **_**(Ω)**	***R***_**CE **_**(kΩ)**	**CPE2-T (μS.s**^***n***^**)**	**CPE2-P (0 <** ***n*** **< 1)**
Pt	26.12 (20.45)	0.71 (3.19)	3.03 (55.78)	0.96 (0.68)
Graphite	24.32 (24.31)	1.03 (1.08)	3.55 (128.10)	0.94 (0.81)
Carbon soot	23.10 (26.84)	0.40 (7.21)	4.92 (31.13)	0.94 (0.73)
Cu_2_S	7.88 (8.15)	0.02 (0.46)	52.64 (18.41)	0.71 (0.84)
RGO	17.62 (17.45)	1.02 (1.83)	10.46 (11.13)	0.82 (0.83)

EIS results of CdS QDSSCs with different CEs under 1,000-W/m^2^ illumination and in dark (shown in parenthesis): series resistance, charge-transfer resistance and impedance values of the constant phase element (CPE).

From the EIS results, it can be seen that the CdS QDSSC with Cu_2_S as CE has the lowest series resistance, *R*_S_. This is reasonable considering the highly conductive brass metal involved in comparison to the usual FTO layer used. *R*_S_ is the resistance corresponding to the transport resistance of the conducting substrate. In this study, charge-transfer resistance at the QD-sensitized TiO_2_/electrolyte interface (*R*_r_) is not discussed as the value is not directly influenced by the choice of counter electrode materials. Under dark condition, the charge-transfer resistance at the CE/electrolyte interface, *R*_CE_ is high in all the cells. When the cells were tested under illumination, the *R*_CE_ value reduced substantially for most of the cells due to more charge transfer taking place in the system. It is observed that the low *R*_CE_ gives rise to higher open-circuit voltage of the cell as seen in the case of QDSSCs with carbon soot and platinum as their CEs. However, this is not the case for Cu_2_S as its photocurrent density is few times lower than that of the cell with platinum as CE. The low *R*_CE_ could be due to the excessive potential bias applied (0.45 V) to the cell as its open-circuit voltage is only 0.28 V. This high potential bias could have provided a more conductive state for the charge transfer. The overall low performance of the cell could be attributed to the low catalytic activity at the Cu_2_S/electrolyte interface which implies a slow reduction rate for polysulfide S_*x*_^2-^ species. For the high-efficiency CdS QDSSCs having platinum, graphite or carbon soot as CEs, the good performance is due to low constant phase element (CPE) values. This translates to low true capacitance at the CE/electrolyte interface which could imply a better electrocatalytic activity.

EIS results for the CdSe QDSSCs are shown in Figure 4 with the corresponding reference data under dark condition depicted in Figure 4a,b. The related series and charge-transfer resistances are tabulated in Table 4. Like in the case of the CdS QDSSC, low *R*_S_ is observed in the cell with Cu_2_S as the CE. In high-performing cells where platinum and Cu_2_S are the CEs, the observed low *R*_CE_ values coupled with low CPE impedance values lead to high catalytic activity at the CE/electrolyte interface. On the other hand, cells with CE from carbon-based materials show high CPE values which result in slower charge transfer through the interface. However, as an exception, *R*_CE_ for cell with carbon soot as the CE appears to be low due to the lower open-circuit voltage compared to the applied potential bias. The *R*_CE_ could be even higher should the applied potential bias is equal to the open-circuit voltage. Contrary to general observation, the cell with RGO as the CE has a lower *R*_CE_ in dark than the value obtained under illuminated condition. We suspect this could be due to inhomogenous dispersion of the RGO flakes on the substrate. As a result, there might be less electrochemical active area for the reduction of polysulfide species S_*x*_^2-^.

**Table 4:** EIS results of CdSe QDSSCs

	***R***_**S **_**(Ω)**	***R***_**CE **_**(kΩ)**	**CPE2-T (μS.s**^***n***^**)**	**CPE2-P (0 <** ***n*** **< 1)**
Pt	26.84 (22.29)	0.28 (0.58)	3.11 (4.57)	0.97 (0.96)
Graphite	28.06 (30.30)	0.88 (0.97)	13.52 (6.15)	0.91 (0.94)
Carbon soot	25.01 (23.22)	0.11 (0.93)	15.17 (10.08)	1.00 (0.86)
Cu_2_S	11.25 (11.28)	0.28 (0.53)	8.09 (3.98)	0.94 (1.00)
RGO	24.48 (22.80)	1.19 (0.71)	8.89 (4.86)	0.86 (0.90)

EIS results of CdSe QDSSCs with different CEs under 1000 W/m^2^ illumination and dark (showed in parenthesis): series resistance, charge-transfer resistance and impedance values of the constant phase element (CPE).

Since the polysulfide electrolyte could impair the platinum CE surface as reported by Mora-Sero et al., the performance of the cell with platinum CE could deteriorate over the long run [27]. Ultimately, the charge-transfer resistance will increase. Therefore, Cu_2_S appears to be a good candidate for CE material for the CdSe QDSSCs. Nevertheless, the high performance as observed in both CdS and CdSe QDSSCs with platinum CE suggests the detrimental effect from polysulfide electrolyte might not be that serious at the early stage of operation. Based on the EIS response, should a multilayered CdS/CdSe QDSSC be prepared, a composite between carbon and Cu_2_S could be the best material for the CE. Similar conclusion has been made by Deng et al. [28]. It is to be noted that the different EIS parameter values obtained for both CdS and CdSe QDSSCs with similar CE materials can be partly attributed to the different choice of electrolytes used as well. Therefore, further optimization is necessary to improve the efficiencies of the cells.

The efficiencies reported in this work are somewhat lower than the values reported in the literature for similar QDSSCs. It should be noted the present study was undertaken with standard TiO_2_ layer sensitized with a single QD layer and standard electrolytes to explore the best CE materials, which resulted in lower efficiencies. A different type of wide band gap semiconducting layer such as ZnO or Nb_2_O_5_ could perhaps produce different results. Nevertheless, the efficiencies of the TiO_2_-based cells can be improved considerably with optimization of all the components involved in the QDSSC and by using passivation layers at the photoanode to reduce the charge recombination losses.

## Conclusions

Low-cost CEs have been prepared from graphite, carbon soot, Cu_2_S and RGO to study their effect on the performance of CdS and CdSe QDSSCs. Carbon-based materials were found to be a good CE material for CdS QDSSCs and such a cell with graphite as CE produced the best efficiency value of 1.20% with the highest photocurrent density. For CdSe QDSSCs, although cell with platinum CE showed a relatively good performance, Cu_2_S could be the alternative choice for CE. EIS measurements on both CdS and CdSe QDSSCs showed that low *R*_CE_ and CPE values for the CE/electrolyte interface are the key criteria for selecting good-performance CE materials. Further optimization of the cell is possible for achieving higher efficiencies.

## Competing interests

The authors declare that they have no competing interests.

## Authors' contributions

HKJ and AKA conceived and designed the experiments. MAC took part in the EIS data interpretation. HKJ carried out the experiments and took part in writing the manuscript. All authors read and approved the final manuscript.
